# User-centered and theory-based design of a professional training program on shared decision-making with older adults living with neurocognitive disorders: a mixed-methods study

**DOI:** 10.1186/s12911-021-01396-y

**Published:** 2021-02-17

**Authors:** Moulikatou Adouni Lawani, Yves Turgeon, Luc Côté, France Légaré, Holly O. Witteman, Michèle Morin, Edeltraut Kroger, Philippe Voyer, Charo Rodriguez, Anik Giguere

**Affiliations:** 1 Laval University, Pavillon Ferdinand-Vandry, Room 2881, 1050 avenue de la Médecine, Quebec, QC G1V 0A6 Canada; 2 CISSS de la Gaspésie – Service externe de gériatrie ambulatoire, 455 rue Mgr Ross Est, Chandler, QC G0C 1K0 Canada; 3 Laval University, Pavillon Ferdinand-Vandry, Room 1323, 1050 avenue de la Médecine, Quebec, QC G1V 0A6 Canada; 4 VITAM Research Centre on Sustainable Health, Pavillon Landry-Poulin, Pavillon Landry-Poulin, Door A-1-2, 4th floor, Room 4578, 2525 Chemin de la Canardière, Québec, QC G1J 0A4 Canada; 5 Laval University, Pavillon Ferdinand-Vandry, room 4211, 1050 avenue de la Médecine, Quebec, QC G1V 0A6 Canada; 6 Quebec Excellence Centre in Aging, St-Sacrement Hospital, 1050 chemin Ste-Foy, Quebec, QC G1S 4L8 Canada; 7 Pavillon Ferdinand-Vandry, Room 3445, 1050 avenue de la Médecine, Quebec, QC G1V 0A6 Canada; 8 Departmentof Family Medicine, McGill University, 5858 chemin de la Côte-des-Neiges, 3rd floor, Montreal, QC H3S 1Z1 Canada; 9 VITAM Research Centre on Sustainable Health, Pavillon Landry-Poulin, Door A-1-2, 2nd floor, Room 2416, 2525 Chemin de la Canardière, Québec, QC G1J 0A4 Canada

**Keywords:** Dementia, Aging, Continuing professional development, Curricular development, User experience, Intervention design, Behaviour change technique, Implementation

## Abstract

**Background:**

We know little about the best approaches to design training for healthcare professionals. We thus studied how user-centered and theory-based design contribute to the development of a distance learning program for professionals, to increase their shared decision-making (SDM) with older adults living with neurocognitive disorders and their caregivers.

**Methods:**

In this mixed-methods study, healthcare professionals who worked in family medicine clinics and homecare services evaluated a training program in a user-centered approach with several iterative phases of quantitative and qualitative evaluation, each followed by modifications. The program comprised an e-learning activity and five evidence summaries. A subsample assessed the e-learning activity during semi-structured think-aloud sessions. A second subsample assessed the evidence summaries they received by email. All participants completed a theory-based questionnaire to assess their intention to adopt SDM. Descriptive statistical analyses and qualitative thematic analyses were integrated at each round to prioritize training improvements with regard to the determinants most likely to influence participants’ intention.

**Results:**

Of 106 participants, 98 completed their evaluations of either the e-learning activity or evidence summary (93%). The professions most represented were physicians (60%) and nurses (15%). Professionals valued the e-learning component to gain knowledge on the theory and practice of SDM, and the evidence summaries to apply the knowledge gained through the e-learning activity to diverse clinical contexts. The iterative design process allowed addressing most weaknesses reported. Participants’ intentions to adopt SDM and to use the summaries were high at baseline and remained positive as the rounds progressed. Attitude and social influence significantly influenced participants' intention to use the evidence summaries (P < 0.0001). Despite strong intention and the tailoring of tools to users, certain factors external to the training program can still influence the effective use of these tools and the adoption of SDM in practice.

**Conclusions:**

A theory-based and user-centered design approach for continuing professional development interventions on SDM with older adults living with neurocognitive disorders and their caregivers appeared useful to identify the most important determinants of learners’ intentions to use SDM in their practice, and validate our initial interpretations of learners’ assessments during the subsequent evaluation round.

## Background

Neurocognitive disorders require making several difficult decisions to ensure older adults remain independent as long as possible, while maintaining their well-being and safety [1]. These decisions may cover daily life management (e.g. being home alone, day care, transportation, home book- keeping), arranging healthcare and support (e.g. diagnosis, medications, home care, domestic help, and respite care), community life (e.g. visiting family, moving house), and representing the person with dementia (e.g. advanced decisions about the end-of-life) [1]. Decisions made by older adults with neurocognitive disorders and their caregivers are generally not only based on clinical information, but also on social considerations (e.g. financial insecurity, availability of community-based organizations, social networks), requiring professionals to expand their knowledge and scope of practice beyond the boundaries of their professions [1, 2]. Since several acceptable alternatives exist for most of these decisions, the priorities of the older adults and those of their family/friend caregivers should guide decision making, together with the scientific evidence on the benefits and harms of the available options [3]. Shared decision-making (SDM) is an ideal approach for supporting older adults and their significant others in making these decisions collaboratively with the interprofessional healthcare team, as SDM is typically used in the context of uncertainty when the person’s preferences are central to the decision [4]. SDM is an approach where clinicians and patients share the best available evidence when faced with the task of making decisions, and where patients are supported to consider options, to achieve informed preferences [5]. Primary healthcare professionals (HCPs) should be trained in SDM and have access to patient decision aids tailored to the needs of older adults living with neurocognitive disorders and their caregivers, as these professionals play a central role in the care and services provided to community-based older adults living with neurocognitive disorders [6–8].

Although SDM can improve the quality of life of patients with neurocognitive disorders and their caregivers [9], decision-making in this context can be challenging. The disabling and neurodegenerative nature of neurocognitive disorders may challenge decision-making by limiting communication with the person as the disease progresses [10–12]. Consequently, neurocognitive disorders are a major risk factor for exclusion from decision-making [11]. Some studies have described the issues and requirements involved with implementing SDM with this population [1, 2, 13–17], but as yet, there have been no studies on the essential characteristics of a training program in SDM for HCPs in caring for older adults with neurocognitive disorders.

Initiatives aimed at increasing the use of SDM by HCPs may comprise training programs, leaflets, financial incentives or email reminders; however evidence remains scarce on their effectiveness to change professional behaviour and improve patient/caregiver participation in decision-making [18, 19]. This project focuses on two strategies to facilitate the implementation of professional training programs in SDM. Firstly, these programs should consider the logistical challenges of attending educational meetings for HCPs, especially those who work in remote areas [20]. In this project, we thus propose a distance training program that is accessible to all professionals, even those living away from the larger centres where continuing professional development activities generally take place. Secondly, we propose using user-centered design to tailor a professional training program to the actual needs and barriers faced by HCPs, as this is a promising approach to ensure that training on SDM leads to actual behaviour change [21, 22]. Indeed, evidence from systematic reviews shows that continuing professional development programs built on well-conducted needs assessments are effective in changing clinicians' behaviours [23]. Training needs assessments have traditionally been achieved through practice audits, questionnaires, environmental scans, or interviews, but the value of user-centered design to this end remains unexplored. User-centered design, which includes design thinking, consists of involving target users in several iterative rounds of evaluations and modifications, to tailor the design of a product to a given task and to the user’s experience [24–27]. In the field of healthcare, preliminary evidence suggests that user-centered design may enhance the implementation in practice of evidence-based information [26, 28, 29] and patient decision aids [30–33].

In this research project, we studied how user-centered design and theory contribute to the development of a distance learning program to support HCPs in implementing SDM with older adults living with neurocognitive disorders and their family/friend caregivers. More specifically, the investigation was guided by the following overarching research question: What are the features of the training program and design strategies that may increase HCPs’ intention to adopt SDM in this clinical context?

### Theoretical framework

**Fig. 1:**
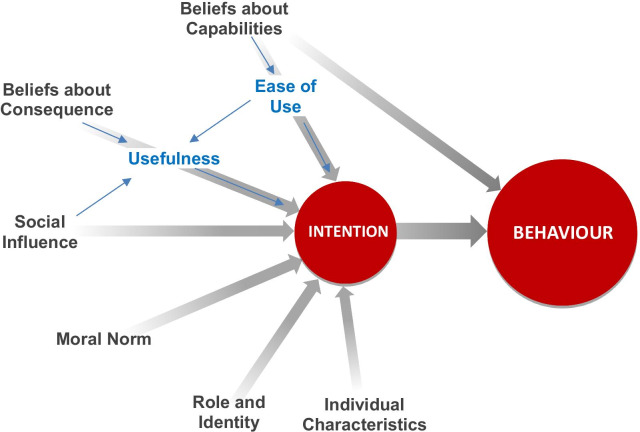
Theoretical model framing the current research

SDM requires the adoption of a diverse set of behaviours by HCPs [34, 35]. This project was thus based on the integrated framework proposed by Godin and al, according to which a behaviour may be predicted by a person’s intention (motivation) to adopt it (Fig. 1) [36]. A person’s intention may, in turn, be predicted by several determinants, including belief about consequences (the perceived advantage or disadvantage of adopting a behaviour), social influence (the perceived social pressure to adopt a behaviour), and beliefs about capabilities (perceived ease or difficulty of adopting a behaviour) [36]. In addition, intention can also be determined by habits/past behaviours and other social and emotional factors, namely moral norms (the feeling of being obliged to adopt a behaviour) and role/identity (beliefs that a person of similar age, sex, or social position should adopt a behaviour) [36]. We then added to this general model the Technology Acceptance Model (TAM-2), which identifies usefulness and ease of use as two specific determinants which could predict users’ intention to use new information technology/information systems [37]. Despite the fact that these two domains are similar to, respectively, the Beliefs about Consequences and Beliefs about Capabilities domains described in the integrated framework, we added them to draw more attention to acceptance (usability and acceptability) of the two studied learning components [38], and thus complement the integrated framework, which is focused on motivation. Use of both the TAM-2 and integrated framework constructs allowed evaluating two dimensions of intention, namely motivation (integrated framework) and acceptance (TAM-2).

## Methods

### Study design and approach

This was a convergent, parallel, mixed-method study in which three HCP subsamples participated in the tailoring of a professional training program. A first subsample helped tailor the first component of the program (the e-learning activity), whereas the second and third helped tailor the second component (a series of five evidence summaries). We used user-centered design approaches for tailoring each component, by improving user experience of the prototypes during several iterative evaluations that each comprised both quantitative and qualitative data collection [39].

### Professional training program on shared decision making

Details on the training program are reported elsewhere [40]. Briefly, the training program included two modalities: (1) a self-directed e-learning activity on SDM, and (2) five evidence summaries, decision boxes (DBs), to support decision-making at the point of care with older adults living with neurocognitive disorders and their caregivers. The self-directed e-learning activity covered the use of patient decision aids to implement SDM. It lasted about one hour, could be completed in several sittings, and was not specific to any clinical area. It was interactive and used narrated slides, videos, and interactive exercises. It also offered an asynchronous forum to discuss any question with an experienced moderator.

The second modality of the training program consisted of a series of five evidence summaries describing the options available to older adults living with neurocognitive disorders who face five important and frequent decisions that we identified in a previous study [41]. These decisions were: (#1) choosing a support option to decrease caregiver burden; (#2) choosing a non-pharmacological treatment to manage agitation, aggression, or psychotic symptoms; (#3) deciding whether or not to stop driving following diagnosis; and (#4) choosing an option to improve quality of life; and (#5) deciding whether or not to prepare a power of attorney (called a “protection mandate” in Quebec, Canada) covering health, property, and financial matters;. The evidence summaries followed the decision box (DB) template, which aims to provide stakeholders with evidence in a format that supports them in SDM [42, 43]. Biefly, these summaries met several of the standards set by the International Patient Decision Aids Collaboration [44]: (1) they described the health condition for which a decision is required; (2) they explicitly stated the decision to be taken into consideration; (3) they described all the options available for this decision; and (4) they described the positive and negative characteristics of each option. Their content was developed from rapid reviews and then revised by clinical experts, as described earlier [45]. The studied summaries are available at www.decisionbox.ulaval.ca/en/ (Series on Older Adults – Problems with Thinking or Memory).

### Population and recruitment strategy

**Fig. 2:**
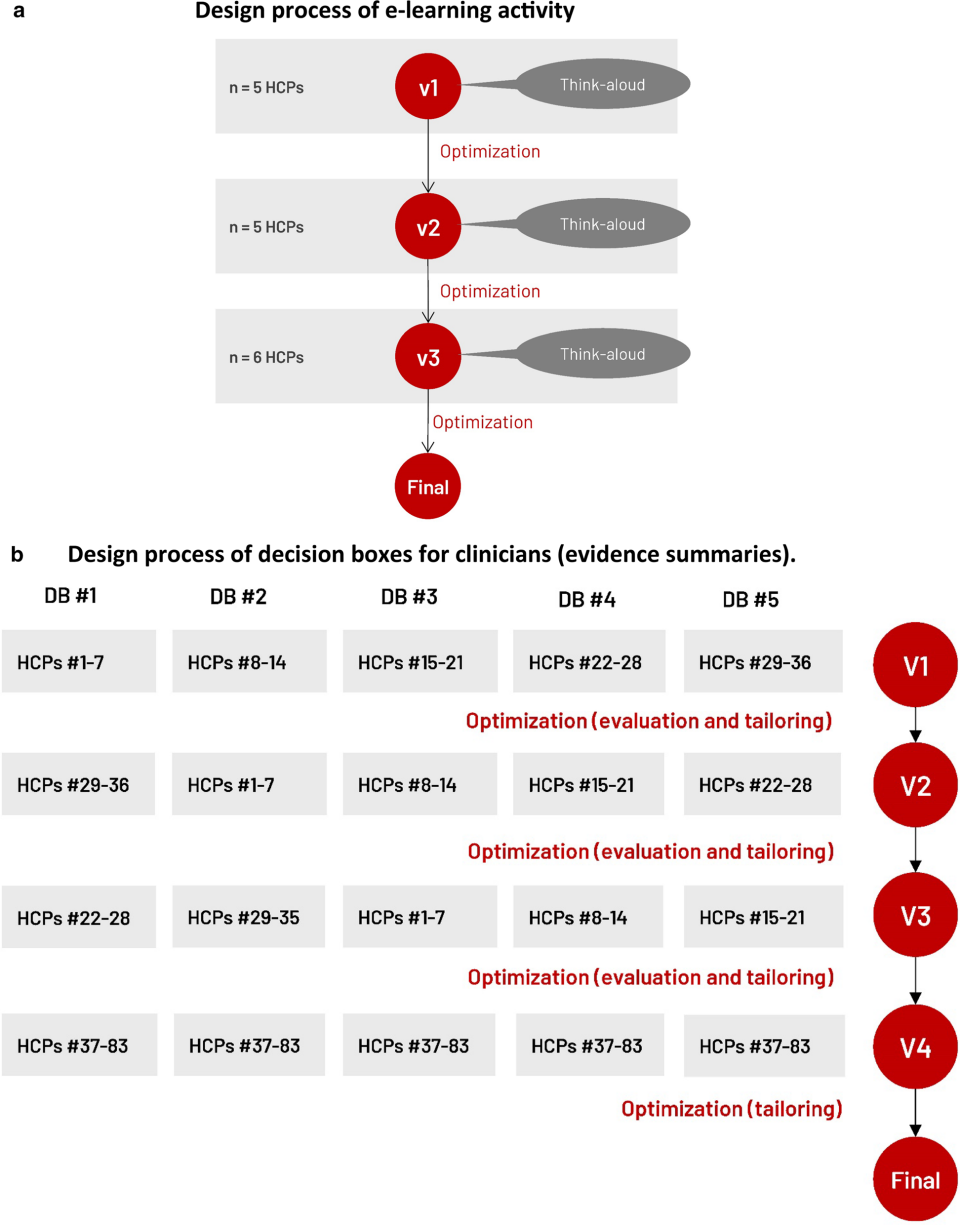
User-centered design process to tailor the training program to user needs. DB #1 = Choosing a support option to decrease caregiver burden; DB #2 = Choosing a non-pharmacological treatment to manage agitation, aggression, or psychotic symptoms; DB #3 = Deciding whether or not to stop driving following diagnosis; DB #4 = Choosing an option to improve quality of life; DB #5 = Deciding whether or not to prepare a power of attorney; HCP = HCP; v = version

We recruited convenience samples of HCPs from any profession (e.g., family physicians, nurses, and social workers) who practiced in family medicine clinics or homecare services in the province of Quebec, Canada. Of the primary care settings invited to participate in the project (46 clinics and 8 homecare services), 20 agreed to participate (16 clinics, 4 homecare services). Figure 2a, b describe the sample distribution for each training component. We carried three or four evaluations/tailoring rounds, with at least five HCPs during each round. These numbers respect human factors validation testing [46].

### Design process of the e-learning activity

#### E-learning activity evaluations

At study entry, all study participants completed a questionnaire on their sociodemographic and professional characteristics. The study participants assigned to evaluate the e-learning activity also completed a questionnaire before and after exposure to the activity, to assess: (1) their preferred role in decision-making using the Control Preferences Scale [47, 48]; (2) their perception of the usefulness and ease of use of the program based on TAM-2 [49]; (3) their level of intention to adopt SDM, and the factors influencing that intention using the CPD-REACTION instrument [50]. To allow calculating means for each of the studied factors, we added three items to the original CDP-REACTION instrument, giving it three items per domain.

These participants also assessed the e-learning activity during a semi-structured think-aloud session that was screen-captured and audio-recorded using Flashback (Blueberry Software). One of two trained moderators (DC, YT) conducted these sessions. After each section of the training, the moderator asked participants about their perceptions on the content and learning strategies used, and recorded any usability issues. At the end of the session, the moderator also asked participants to comment on the main benefits and inconveniences of SDM, which allowed us to describe the factors influencing participants’ beliefs about the consequences of using SDM, one of the components of the theoretical framework guiding this work (Fig. 1).

#### E-learning activity tailoring

Inspired by Susan Michie’s mapping approach, we modified the e-learning activity by adding/enhancing behaviour-change techniques targeting the factors likely to limit the adoption of SDM at each round [51, 52]. To this end, we initially imported the transcripts of the think-aloud sessions as source documents using NVivo coding software (QSR International, version 12). Two researchers (DC, AMCG) conducted a deductive/inductive thematic qualitative analysis of the factors likely to limit adoption of SDM [53]. More specifically, we initially used the theoretical domains framing the questionnaire as themes, and then added new themes as needed. We identified the weaknesses and strengths of the e-learning activity within each theme. We interpreted qualitative and quantitative data together and, following this analysis, the coders (DC, AMCG), moderators (DC, YT), principal investigators (AMCG, FL), and a human factors expert co-investigator (HOW) discussed the functionality of the tutorial and modifications to improve functionality and modify the program accordingly.

### Design process of the decision boxes (DBs)

#### Decision boxes evaluations

The study participants assigned to evaluate the DBs received emails with a link to access a web-based questionnaire to evaluate each DB, at a rate of one per week for five weeks. The DBs were also available on a website. The questionnaire served to (1) explain the purpose of the DB; (2) allow participants to access the DB under evaluation by clicking on a link; and (3) ask a series of questions about what they thought of the DB.

Congruent with the theoretical approach adopted, we used two questionnaires to assess the psychological construct ‘intention’ and its potential determinants. We used the CPD-REACTION questionnaire, which is based on the integrated framework described above and was created as a routine assessment of the impact of continuing professional education on practice [50]. We also assessed the usefulness and ease of use of the DBs, based on the TAM-2 [49]. For each of the CPD-REACTION and TAM-2 items described above, participants rated their perceptions using Likert items ranging from 1–7 (with 1 being the lowest). If ratings fell below four for any item, we then asked the HCPs to explain the reason for their rating in an open-text field, to allow us to understand the barriers they perceived to adopting SDM. At the end of the questionnaire, an open-text field also invited participants to include any additional feedback on the DB.

We sent two weekly reminders to participants who had not completed their evaluations.

#### Decision boxes tailoring

After each round, we used descriptive statistics to summarize participant ratings. We also imported the qualitative comments made in the open-text fields of the questionnaire into a specialized software program (NVivo), and two researchers (MAL, AMCG) analyzed them using a thematic deductive/inductive qualitative analysis approach [53]. We initially used the theoretical domains framing the questionnaire as themes, then added subthemes as needed [53]. We identified the weaknesses and strengths of the DBs within each of the theoretical domains, then broke them down further into emerging themes, to describe the weakness or strength. We resolved any disagreement by consensus between the two researchers.

An interdisciplinary expert panel subsequently met to review the qualitative and quantitative findings, and identified strategies to improve the DB, so as to limit the identified weaknesses. The panel consisted of a graphic designer (JB), a human factors engineer (HOW), an epidemiologist (MAL), and four knowledge-translation researchers (AMCG, HOW, MAL, DC). The experts started by prioritizing each of the problems uncovered. Then, we determined the most appropriate solutions by considering the magnitude, frequency, and severity of these problems, and modified the DBs so that HCPs could explain the pros and cons of health options, as understood from the DBs, to patients and their caregivers.

We used the same evaluation/tailoring process again in two more rounds, with new participants each time.

### Final quantitative analyses

We used descriptive statistical analyses to summarize participants’ characteristics, their interest in each DB topic, their level of intention, and the potential predicting determinants of their intention. We used SAS (Version 9.4, copyright 2002–2012, SAS Institute Inc.) to conduct these descriptive statistics, and a significance level of 0.05.

### Integration of quantitative and qualitative findings

After the entire series of evaluation/tailoring rounds, we integrated the quantitative and qualitative findings to generate conclusions on the factors influencing intention, and on the changes in users’ intention as the rounds progressed.

## Results

### Study population

**Fig. 3:**
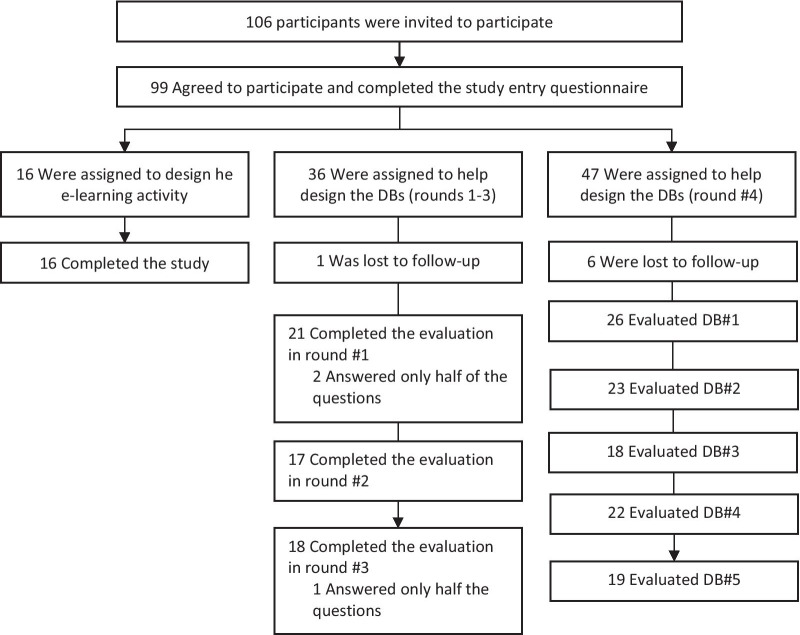
Description of participants’ samples in each of the three sub-studies. DB = decision box

One hundred and six HCPs from 20 clinics and homecare services located in 13 cities, agreed to participate in this study. A first cohort of 16 HCPs was assigned to help design the e-learning activity, a second cohort of 36 different HCPs helped design the DBs in a three-round evaluation process, and a third cohort of 54 helped design the DBs by providing comments in a fourth evaluation round. These cohorts are represented in Fig. 3.

In the three cohorts, 78% of participants were women, 60% were physicians, and 57% had fewer than 20 years of practice (Table 1).

**Table 1:** Characteristics of study participants

Participant characteristics		Frequency (Total n = 99)	
		n	%
Age (years)	Under 30	16	16.1
	30–39	27	27.3
	40–49	27	27.3
	50–59	23	23.2
	60–69	5	5.1
	Missing	1	1.0
Gender	Female	77	77.8
	Male	22	22.2
Profession	Physician	59	59.6
	Nurse	18	18.1
	Social worker	13	13.1
	Occupational therapist	6	6.1
	Pharmacist	1	1.0
	Physiotherapist	1	1. 0
	Nutritionist	1	1. 0
City sizes of practice area ^a^	Small city	35	35.4
	Medium city	7	7.1
	Large city	57	57.6
Years of practice	< 10	35	35.4
	10–19	21	21.2
	20–29	27	27.3
	30–39	10	10.1
	40–49	3	3.0
	Unsure	3	3.0

^a^City size < 1,000 = rural; 1,000–29,999 = small; 30,000–99,99 = medium; > 100,000 = large (Statistics Canada, 2011)

### Tailoring of the training program

**Fig. 4:**
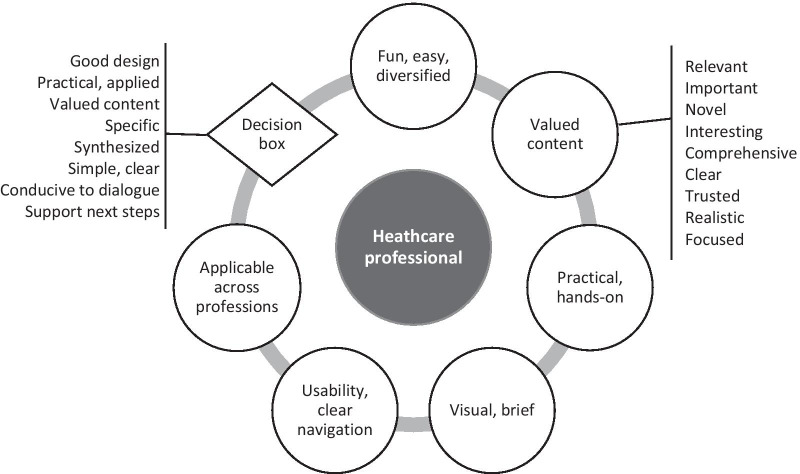
HCPs’ experiences of the training program

The data that was used to tailor the training program is presented in this section and summarized in the Fig. 4. In the following two subsections, we successively report participants' perceptions of the e-learning activity and decision boxes, and the strategies used to solve the problems discovered during the evaluation. In the third subsection, we present how participants' perceptions of SDM supported the tailoring of the training program.

#### Tailoring of the e-learning activity based on user experiences

Participants expressed their appreciation of the content and design of the e-learning activity on numerous occasions, and in every section (Table 2). They especially liked the narrated slides, the quiz that informed them of actual patient numeracy levels, and the film depicting a simulated patient encounter during which SDM is implemented.

**Table 2:** Strengths of e-learning component of the program

Themes and subthemes		Illustrative citation
**Valued content**		
Introduction module (overview of the activity): clear, relevant, comprehensive, and trusted		“It was really comprehensive, and the video was very helpful.” (Nurse #16, Round #3)
Shared Decision-making module: interesting, relevant, novel, important, clear, uses useful examples, and use of an appropriate terminology for the “Watchful Waiting” option		“What’s interesting is the fact that it doesn’t necessarily apply to every decision. You know, I would have thought they would say: ‘Shared decision-making must be the focus of every decision made with your patients.’ […]. I think it’s good it’s not being presented as a dogma where you don’t have the right to make a decision.” (Physician #7, Round #2)
Risk Communication module: relevant, appropriate, allows understanding patient perceptions of numbers, and interesting information on the level of evidence		“You have to realize that people don’t all have the same level of education, and the way I understand something may not be the same way my patient sees it. It makes you realize things; it makes you question yourself and your competencies.” (Physician #3, Round #1)
Patient Preferences module: foundational to shared decision-making, interesting, useful prompt of the five key behaviours in the SDM process, and valued content on the climate conducive to dialogue		“I found this part really important because, in my opinion, it’s the crux of the approach. What I mean is that if we don’t approach the matter properly with the patient, then we’re not respecting their values and preferences, and we’re missing something. So this is something that’s really meaningful to me.” (Social worker #9, Round 2)
Implementation module: interesting, valued exercise on the barriers/myths in implementing SDM, and overall supportive of implementation of shared decision-making		“I like it because it brings us back a bit to the pre-test with any preconceived notions we may have had, and it debunks them. It provides solutions. Honestly, I think it’s a really good section.” (Physician #13, Round #3)
**Instructional design supportive of learning**		
Narrated slides: appropriate learning modality, changes of narrators between modules are appreciated, images and visual design appreciated		“I think it’s good because it’s interactive, there’s someone talking to you, explaining it to you, with slides. It’s easy to understand. They don’t go into long-winded explanations.” (Nurse #1, Round #1)
Film displaying implementation of shared decision-making: good synthesis, hands-on, a realistic example of SDM application and of decision box use, the demonstration of best practices is valued (as opposed to the demonstration of bad practices), the interactive exercise after viewing of the film is appreciated to allow critical appraisal of the behaviour displayed		“As a matter of fact, it’s probably the most interesting part because in the beginning it’s all theory, and then, it’s more about practice, so in my view, it’s extremely relevant.” (Physician #5, Round #1)
Quiz on numeracy: allows becoming aware of the challenges of risk communication, requires reflection, fun, and appropriate learning modality with questions-answers		“Sure, it puts us to the test! [laughs]. But it makes you realize that we all have different skillsets, including comprehension… “ (Physician #3, Round #1)
Exercise on the barriers/myths related to shared decision-making: appropriate learning modality as it is interactive, realistic in that it describes typical myths propagated by HCPs		“It’s really interactive. I like that. Not too much reading. Point form. I just retain information better that way.” (Physician #13, Round #3)
Introduction video: clear, brief, and visual		“It puts things into context nicely. I really like the part where she shows what the website looks like and the different features of it.” (Physician #13, Round #3)
Personal stories using videos with avatars: integrate much information, and nice format		“I’ll remember the little avatar guy. It’s a good memory aid. Like pop-up reminders: ‘Oh yeah, I need to take out such and such a thing’ or ‘That reminds me of my patient’… because we have no shortage of documents, about antibiotics, about this or that… I found it interesting, the use of a visual aspect like that… it’s a bit ‘lighter’” (Physician #5, Round #1)
Overall program: allows bringing everyone to the same level, appreciated integration with the directory of decision boxes, valued the diversity of learning modalities, topics well-illustrated, appropriate length, clear navigation throughout program		“The interactive features you put in were not too long. They were at just the right time, and it mixed up the rhythm a bit. I thought it was really good.” (Physician #13, Round #3)
Critical appraisal exercise: appreciated that some part of the text is highlighted, well-synthesized, clear, novel information, appreciated interactivity, challenging		“Of course, I’m really on the ground and very hands-on. So, when I have to deal with research findings and scientific language, you lose me a bit. But what I found good was that you put the main message in bold. You don’t have to read the whole statement. I just read the part in bold and I got the gist of it.” (Social worker #15, Round #3)
To Learn More section: appreciated		“It’s really good because there are always people who want to learn more or investigate further. If there’s something they didn’t understand, they can go directly to the sources, which I thought was good.” (Social worker #15, Round #3)

User-centered design initially allowed discovering several weaknesses of the e-learning activity. The approach then made it possible to remedy these weaknesses and then evaluate the impact of the changes made during the next evaluation round. For example, participants mentioned several elements lacking clarity, either in terms of the training component or the content (Table 3). We therefore systematically corrected the most specific issues (e.g. an acronym is undefined, a source is not cited) and they were not mentioned again by the participants in the following rounds. However, for the more general elements lacking clarity (e.g. the availability and content of the DBs, the main principles of SDM), we added an introductory video after Round #2. In Round #3, participants all mentioned appreciating this introductory video.

**Table 3:** Weaknesses of e-learning component of the program

Themes and Subthemes		Illustrative citation
**Lack of clarity**		
Training modality: more instructions needed on what is a decision box and how to access them, exercise is unclear		“At first I wondered what the decision box was. But in reality they’re tools. It could be a good idea to mention what it is, because, in the beginning, I didn’t know what it was. I thought it was something optional, but really it’s not.” (Physician #5, Round #1)
Unclear content: important information should be highlighted or bolded, more precisions required, missing information		[Note from the author: The participant is discussing the critical appraisal exercise] “I don’t know if people will understand it. They aren’t necessarily simple terms. It’s not the kind of thing you see every day. Especially not for clinicians who don’t do much research. For those who’re in journal clubs, it’s usually about interventions and relative risks… that sort of thing.” (Physician #14, Round #3)
**Usability**		
Browsing issues between pages or sections: not intuitive enough, need to clarify how to move to the next section after completing an exercise, access to optional content is unclear		“I didn’t know where to click. I’m not very tech-savvy. I wasn’t sure what you meant by ‘Target Clientele.’ But now I understand that it’s part of the introduction. Maybe you could say ‘Introduction’ instead of ‘Target Clientele?’” (Physician #11, Round #3)
Issues with clickable elements: references within narrated slide shows should be clickable, element dispositioned, some slides should be reordered, confusions between buttons to move to the next page		“Click here to begin reading […]. The arrow is in the wrong place.” (Physician #7, Round #2)
Elements too small: images, narrated slide show		“It would be good to make the button more visible. I’m imagining someone who’s not very comfortable with computers… you know, sometimes, depending on your screen, it can be harder to see.” (Physician #5, Round #1)
**Irrelevant or of less value**		
Content unrealistic: statin example irrelevant for people at low risk of cardiovascular diseases, the film displays a simulation, not reality		“In reality, I’m not sure people would be inclined to talk about taking statins with a patient who’s low risk. I understand; I’ve seen the studies showing there may be some benefits, but at the same time, with figures like that, it’s not necessarily a case where I would use shared decision-making.” (Physician #14, Round #3)
Irrelevant for specific audiences: clinical information not applicable to all professions, information more relevant for older HCPs, information les useful to experienced/less experienced physicians		“This part is a bit more about the medical side, and that one could be more for social workers or others. Sure, there are things that are a bit less relevant for me that I can still explore further, but you know, when I see certain things, I’m more likely to just refer them… “ (Social worker #9, Round #2)
**Inappropriate learning strategies**		
Vignette with avatars displaying patient counseling strategies: not realistic enough, robot-like, childish, information is too theoretical, optional content		“When you’re a clinician and you watch that, you see interview techniques with the patient. The problem with the video is that the avatars don’t have any intonation. When we’re taught how to communicate with our patients, we’re taught what intonations to use. But in this case, there is no intonation. They always speak in exactly the same tone of voice.” (Physician #5, Round #1)
Critical appraisal exercise (Cochrane review on the impacts of patient decision aids): information layout should be improved, boring, reading is less appreciated as learning strategy		“I hate reading studies, especially studies in English! I get confused with the words […]. Even in French, I don’t like it, but in English it’s even worse! I’m just not interested. Especially with the way it’s presented here. There are too many figures and, in any case, it really doesn’t interest me.” (Physician #2, Round #1)
Film: unrealistic. communication of statistic still confusing		“The fact remains that it’s a simulated interview. You know, it’s not reality. It looks nice, but in real life, it’s not often like that.” (Physician #6, Round #2)
**Too long:** the whole program is too long, some sections are too long, the film is too long, some exercises are too long		“I think it’s a bit long in places. The content on decision aid tools was perhaps a bit long. There were several slides presenting the tools, and I figure people will be able to understand it easily enough. After all, it’s aimed at HCPs.” (Physician #7, Round #2)
**Dry:** relevant but dry (critical appraisal exercise, and content on evidence appraisal)		“When you get to this part, it shifts from visual mode, which is easier to follow, to a more purely informational mode, with words in English and all that, and lots of text. […]The information is clear enough, but it’s perhaps a little harder to find it. It’s not as simple as with the videos.” (Physician #5, Round #1)
**Technical issues:** video does not work, difficulties moving to the next slides, feedback does not show, loosing the Internet connection		“The video feedback doesn’t show up, only text.” (Social worker #15, Round #3)
**Boring:** first part is boring, reading is boring, narrated slide shows are boring, lack of animation		“I’m a very visual person. When there are long stretches of narration only, I just switch off.” (Physician #13, Round #3)
**Terminology issues:** acronyms should be defined, some terms that are too strong, unclear terminology, what do “option” and “numeracy” mean?, avoid the term “choosing the option of doing nothing”, some terms are difficult to understand		“A list of available health options… I can’t picture exactly what that means.” (Physician #3, Round #1)
**Risk communication seems challenging**		“I don’t always recall all the probability percentages. In a single day. I might see fifteen patients, all with different problems. I can’t always remember off the top of my head what the probabilities are.” (Physician #2, Round #1)
**Optional content**		
Overall description of the program, program design team,		“You know, we’re so pushed for time that we really want to get straight to the point which, in this case, is the training. I get it that it’s really well done and all that, but I would still try to condense it a bit more.” (Physician #5, Round #1)
GRADE quality of evidence assessment: outside the program’s scope, information that is already known, covered in other programs		“I found it less relevant for shared decision-making, which made it less interesting.. […]. If I wanted to watch a webinar on shared decision-making, I wouldn’t want to watch that.” (Physician #8, Round #2)
**Incomplete, missing information:** missing sources, specific details missing, missing feedback on some exercises, abbreviations or acronyms are not defined		“I don’t know if it’s feasible, but it would have been fun to have a DB in hand to follow along at the same time as the training session.” (Physician #8, Round #2)
**Redundant content:** could be shortened, slightly too repetitive		“At one point they give a lot of examples. Maybe there are some things that could be removed.” (Physician #7, Round #2)
**Informational or typographic error**		“*’Are you sure about which choice is best for you?*’ Knowing some of my elderly patients, I think this question could destabilize them or make them feel less confident in their decision. I wouldn’t have worded it that way. I would have validated that that is the decision they want to make, but I find it a bit strong to use words like ‘certain’ or ‘best.’ Personally, I would have taken a gentler approach in that sense.” (Physician #11, Round #3)
**Patient Preferences section too short:** more communication tips should be offered		“Not everyone in the medical field has good patient communication skills, so I think you could expand this section a bit.” (Social worker #15, Round #3)

Several other types of issues were corrected systematically, such as usability issues, or features perceived as irrelevant or less valuable such as the videos with avatars, the introduction, or the training module explaining the evaluation of evidence quality. We either removed these elements, or made them optional for people interested in learning more.

We received several comments on the inapplicability of some of the examples across professions. Therefore, in the final version we diversified the professions displayed in the examples offered.

The activity required participants to extract the benefits and harms of patient decision aids from a scientific abstract of the Cochrane systematic review on their impact. Participants found the exercise difficult. We chose to try to improve the format of the exercise first, to make it easier. We therefore simplified the text of the abstract as much as possible, added pictograms and smiley faces to help identify the benefits and harms, and translated it into French, to further understanding of the information. We received no further negative comments from participants on this exercise thereafter.

#### Tailoring of the decision boxes based on user experiences

Participants reported general interest in the topics covered in the decision boxes (DBs), with an overall mean interest of 80% (± SD 11%). The DB perceived as least interesting concerned the power of attorney (67% ± SD 29%), and the one perceived as most interesting covered non-pharmacological treatment to manage agitation, aggression, or psychotic symptoms (88% ± SD 11%). Participants mentioned that they liked the visual design of the DBs, because it facilitates their use in practice, especially the tabular format presenting risks and benefits/harms (Table 4). Several participants also expressed their satisfaction with the informational content of the DBs, especially the information about the options. Some mentioned that the DBs helped make them aware of the options, or found that the options were relevant. One person appreciated the fact that the DB was available to support older adults in realizing, on their own, that their driving might be dangerous. They thought this might help maintain their relationship with patients.

**Table 4:** HCPs’ perceptions of the strengths of the Decision Boxes

Themes and Subthemes		Illustrative citation*
**Visual design**		
Good visual design		“Very nice tool with an excellent visual presentation.” (Physician #22, Round #1, DB #3) ; “Well-designed tool. Easy to use.” (Physician #31, Round #2, DB #3)
Tabular format: tables are very clear and visual		“Very clear, visually appealing tables. The *Confidence in these results* pictograms could be a bit more visible (black dots rather than a cross? Bigger circles?). The presentation page (page 1) is a bit dry to read, but essential for explaining the goal.” Physician #12, Round #1, DB #1)
Balance between benefits and harms is useful “Nice layout of benefits vs harms.” (Physician #74, Round #4, DB #2)		
**Informational content**		
Value of the information about the options		“The box presents some very interesting options.” (Physician #32, Round #3, DB #1)
Raises awareness about certain options		“The role of case manager no longer exists in many CLSCs. Highly relevant and appropriate for our family caregivers who are unsure or unaware of which resources to turn to. I will definitely use it.” (Social worker #12b, Round #4, DB #1) ; “Great idea for improving our client service.” (Social worker #48, Round #4, DB #4)
Information allows HCPs to keep up-to-date and to empower patients		“Nice tool that allows us to be more professional and access up-to-date knowledge. Also enables us to show that we respect the client’s values. Helps empower them.” (Social worker #48, Round #4, DB #1)
A tool to help older adults realize themselves their own risks		“Very interesting toolbox for guiding and helping patients realize on their own that their driving may not be safe, instead of having the impression, as a doctor, that you are taking away their license and their autonomy. It helps maintain the quality of the therapeutic relationship.” (Physician #36b, Round #4, DB #3)
Useful to remind me of something I already know		“The information in the box will be helpful for refreshing my memory on the various power of attorney options.” (Physician #31, Round #1, DB #5)
**Implementation of SDM**		
Useful to adopt a shared decision-making approach in their practice		“I have never (or rarely) discussed stopping driving with a patient based on the risks and benefits to the patient. Rather, I tried to test the patient's skills through tests without necessarily dwelling on his understanding of the risks of driving. Participation in this study will make me more likely to approach the risk-benefit aspect with the patient in the future.” (Physician #12, Round #3, DB #3)

*DB #1 = Choosing a support option to decrease caregiver burden; DB #2 = Choosing a non-pharmacological treatment to manage agitation, aggression, or psychotic symptoms; DB #3 = Deciding whether or not to stop driving following diagnosis; DB #4 = Choosing an option to improve quality of life; DB #5 = Deciding whether or not to prepare a power of attorney.

The limitations reported by study participants on the decision boxes allowed enhancing them to improve user experiences. Some of the modifications made were quite extensive. For example, several participants reported lower beliefs about their capabilities to use the DB to explain the pros and cons of health options to patients, because of the lack of accessibility to the services described in the DB (Table 5). We judged this a critical flaw, as if it remained unresolved, HCPs would not use the DB and adopt SDM. Therefore, to resolve this aspect, we added a section with a list of contact information for professional and community services available to implement the options offered in the DB, such as massage therapy, music therapy, and physical activity. Another critical flaw was that several participants perceived that they might not have enough time to use the DB. We therefore added some content describing the situations where SDM should be prioritized. We also added information about the possibility to delay decision-making to a subsequent consultation, and thus limit the time needed to go through the complete SDM process.

**Table 5:** HCPs’ perceptions of the weaknesses of the Decision Boxes by theoretical domain.

Theoretical Domain, Weakness		Illustrative citation*
**Intention**		
I have no intention of changing my day-to-day approach.		“The information in the decision box will be useful to me to refresh my memory on the different protection regimes but I do not believe that I will change my way of doing things from day to day.” (Physician #31, Round #1, DB #5)
**Belief about consequences of using the DB; Usefulness of the DB**		
The DB is not well adapted to every patient, nor is it adapted to every situation		“The facts were generally known to me, even though I did not have precise statistics. In addition, each case is different, and I do not believe that applying this box will allow me to better target the necessary interventions.” (Physician #19, Round #2, DB #3)
DBs do not add any value to clinical practice		“We understand that quality of life is the main objective, but the toolbox, although interesting, is heavy and brings nothing more to the clinical level.” (Physician #31, V3, DB #5)
DBs are not helpful to announce my own opinion/recommendation		"What is most difficult is to announce our opinions when they are very divergent from those of the patient or the family, and I do not believe that the decision box helps us." (Physician #19, Round #2, DB #3)
Statistics are useless		"The DB, while interesting, is cumbersome and brings nothing more to clinical practice."(Doctor #31, Round #3, DB #4)
The same information can be offered informally without presenting the DB		“There are many tools for different aspects of medicine, and the use of concrete tools is difficult, but the idea conveyed by these tools can be transmitted verbally to patients informally through other information.” (Physician #9, Round #1, DB #5)
Information is harmful to interprofessional collaboration		"I was surprised by the statistics that 1% to 3% of therapists commit sexual acts that could hinder the process of care. Considering the low proportion of men, the percentage of therapists with deviant behaviours seems to me to be high. I very much doubt this statistic. As I am a man, I find that this statistic might affect referral and the confidence of the doctor towards the therapists." (Social worker #69, Round #4, DB #1)
Already aware of this information		“I was aware of the facts, but not of the exact statistics.“ (Physician #19, Round #2, DB #3)
**Belief about capabilities: I might not be able to use the DB to present the pros and cons of health options to patients because…**		
… it is challenging to access the services offering the health options described in the DB		“Accessibility remains a problem in many regions of Quebec. These data have been collected to facilitate discussion, but the bulk of the work that will ultimately increase the patient's quality of life is not the DB, but the long-term accessibility to this type of service.” (Physician #32, Round #2, DB #4).
…it is challenging to present statistics		"When patients and their family caregivers are in our office, talking about statistics is unthinkable. It is already very difficult to only talk about the reality of everyday life." (Nurse #14, Round # 2, DB #1)
…I don’t have time		‘’Office days are becoming heavier and heavier and it is now possible to use the services of our paramedical staff to convey more complete information so we can focus more on clinical tasks.” (Physician #31, Round #1, DB #5).
...I don’t know how to access the DBs.		“Access to the DB should be facilitated.” (Nurse #15, Round #2, DB #4)
**Ease of Use**		
The DB uses unfamiliar jargon or terminology		"The terms used are legal terms and it's a tough jargon for me." (Physician #20, Round #2, DB #5)
The information is too dense and should be synthesized		"The presentation is too dense and too complex." (Physician #2, Round #1, DB #1)
The information is difficult to understand		“I found the latest data on using vs stopping medication confusing. In the Harms section, it is hard to interpret what you’re trying to show.” (Physician #32, Round #1, DB #2)
Usability of the DBs could be improved "The Pictograms for the *Confidence in the results* section could be a little more visible. Could black dots be used rather than crosses? Could the circles be enlarged?" (Physician #12, Round #1, DB #1)		
**Social influence: My colleagues might not use the DB to present the pros and cons of health options to patients because…**		
…experience is more valuable than the information in the DB		"I believe that professional experience allows us to properly target the discussions to be had with patients and their families." (Physician #19, Round #2, DB #3).
...the DB is long and complex		"Too complicated, too long, and does not match the reality of work." (Nurse #14, Round #2, DB #1)
...some information is irrelevant		"There is too much irrelevant information in the tables." (Physician #30, Round #3, DB #1)
**Role/identity**		
This topic is irrelevant to my professional role		"I think my colleagues at the clinic usually have the same habits as me and refer to our social worker for this part of the job." (Physician #31, Round #1, DB #5)

*DB #1= Choosing a support option to decrease caregiver burden; DB #2= Choosing a non-pharmacological treatment to manage agitation, aggression, or psychotic symptoms; DB #3= Deciding whether or not to stop driving following diagnosis; DB #4= Choosing an option to improve quality of life; DB #5 = Deciding whether or not to prepare a power of attorney.

In some cases, we chose not to attempt to resolve the issues raised. For example, some participants felt the statistics were too hard for patients and their family caregivers to understand. Since probabilities in numerical formats are required to understand risks, we chose not to change the risks presentation in the DBs. Instead, we modified the e-learning activity by adding a module describing best practices to communicate risks to people with lower numeracy skills.

Similarly to the e-learning activity, many of the participants’ comments were specific and easily addressed. For example, participants mentioned that the information was too dense, the terminology challenging, or some usability issues. We therefore adjusted the content of the DBs to reduce their length and complexity, and thereby limit the time needed to understand them, without compromising their meaning. We kept the use of jargon to a minimum, and added a glossary where we were unable to find simplified terminology. We also synthesized the scientific information on the pros and cons of the options in summary tables where possible. When there were more than two options for a given clinical situation, we added a table setting out the potential options for the decision at hand on the first page, including the estimates of the probabilities of impacts for each option, as well as the corresponding page detailing the impacts.

A few physicians reported that the topic covered by DB #5 (Power of Attorney) was irrelevant to their professional role. In an earlier Delphi study [41], we identified a need in primary care practices for decision support regarding this topic. We consequently attempted to improve DB #5 by making some of the legal aspects of the power of attorney easier to understand so that HCPs, especially physicians, can take ownership of the content and become more comfortable discussing it with their patients.

#### Tailoring content based on participants perceptions of SDM

We explored participants’ beliefs about consequences, or usefulness of SDM process after completing the e-learning activity. Participants’ descriptions of the benefits and inconveniences of SDM (Table 6), were very useful to appraise participants knowledge after training, and tailor the content to improve knowledge. Several of these comments pointed to known barriers to adopting SDM. We added specific content to the existing modules to address each of these concerns. Overall, these comments led us to describe several strategies for adopting SDM in diverse clinical situations, for example, where time is limited, when there is an emergency and a decision cannot be delayed, when the patients’ preferences go against those of the professional, when risk is low, or when there are several decisions to be made. We also clarified the role of HCPs in situations where the patient’s choice appears contrary to public health recommendations.

**Table 6:** Participant perceptions of the pros and cons of adopting shared decision-making (SDM) as assessed using open-ended questions after participants’ exposure to the e-learning activity

Themes and Subthemes
**Pros of SDM**
SDM allows patient-centered care by adapting practice to each patient
SDM may increase patient adherence to treatment
SDM increases patient’s perceived control over their health and satisfaction with care
SDM leads to clinician and patient satisfaction about the decision
SDM respects ethics
SDM may be challenging but pays in the long run
SDM decreases clinician’s perceived decisional burden
SDM structures the discussion with the patient
SDM improves patient’s perception of being understood
SDM allows clinician to provide patient support with a human approach
SDM helps establish trust
SDM enhances patient comfort with the decision; limits regret
SDM allows for improved risk perception by patients
SDM does not increase duration of the clinical consultation
SDM is useful in uncertain situations
**Cons of SDM**
SDM takes time and is therefore not appropriate in emergency situations
SDM may depend on patient’s personality or health condition
Applying SDM is challenging without training or tools that provide access to probabilities
Patient’s preference could contradict the clinician’s recommendations
SDM is not appropriate when risk is low
SDM is incompatible with phone follow-ups
SDM cannot be applied to every question the patients have, since they are too numerous
Patients might ask clinicians to make the decision even if SDM principles are applied
Patient preferences could contradict public health recommendations
Applying SDM can be difficult for decisions on goals of care
Applying SDM may be challenging when there are several equivalent options
SDM could lead to multiple medical consultations
SDM is not applicable in cases of immediate treatment

Some of the participants’ comments after reading a decision box also point to a lack of knowledge about SDM, for example that DBs are of little use when stating their opinion or making recommendations (Table 6). To improve understanding of the SDM approach, we added an introductory paragraph to all DBs, entitled “What's this document for?” which described the general SDM approach. We also added patient stories to most of the DBs, usually demonstrating an encounter between a patient and a clinician, to demonstrate the value of seeking patient priorities. The stories were created from testimonies gathered online and were validated by the expert panel. These strategies proved effective, as we received no more comments suggesting that SDM might not be well understood after these changes.

### Changes in the level of intention as the rounds progressed

#### E-learning activity

Visual examination of the quantitative descriptive results suggests that there was no change in participants' intention to adopt SDM after their participation in the e-learning activity. Intention was relatively high and stable across the three rounds, with a mean level of 6.8, on a scale ranging from 1 (low intention) to 7 (high intention) (Additional file 1). Mean levels of potential factors influencing intention were also relatively high, ranging from 5.6 to 7.0, on a scale ranging from 1 to 7.

#### DBs: Levels of intention and the determinants of this intention

**Fig. 5:**
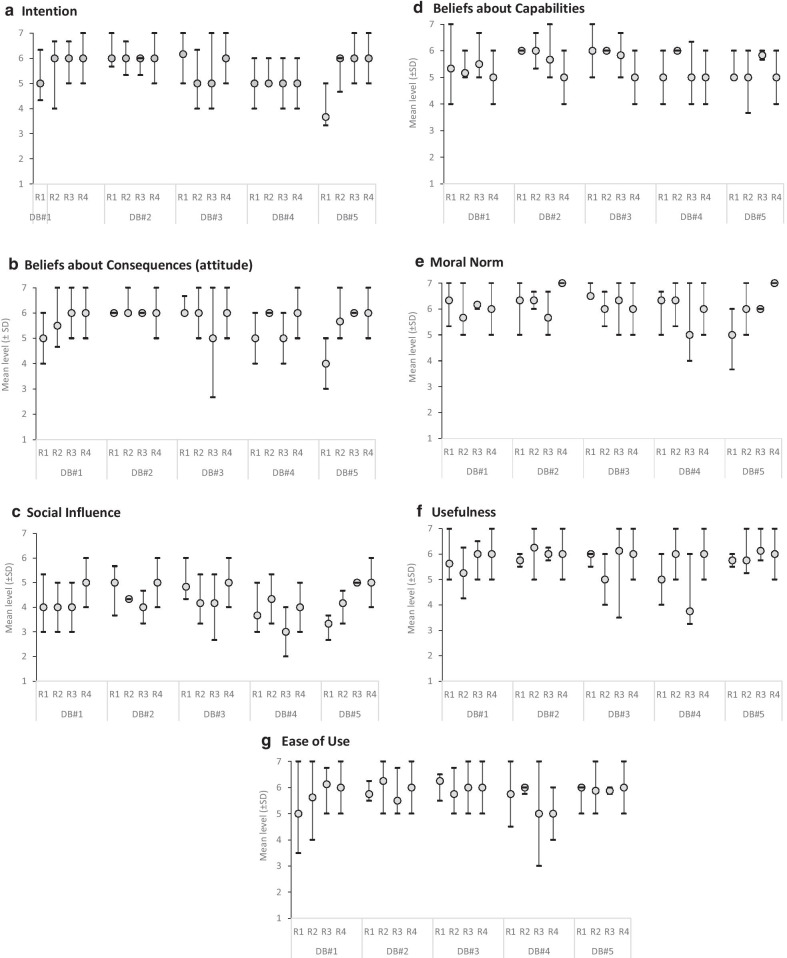
Mean levels of participant intention and its determinants after their review of each decision box, at each round. Legend: DB = decision box; R = round; SD = standard deviation

Participants’ level of intention to use what they learned through the DB to explain the pros and cons of health options to patients was also relatively high and stable across the three rounds, with a mean level of 5.7 for all the DBs (Fig. 5a, Additional file 2). Mean levels of potential factors influencing intention were also relatively high, ranging from 4.5 to 6.4, on a scale of 1 to 7. Participants’ level of satisfaction was generally positive, with mean values of 4.2 on a scale ranging from 1 (low satisfaction) to 5 (high satisfaction) (Fig. 5b–g).

## Discussion

In this study, we used theory-based and user-centered design approaches to tailor a professional training program on SDM to the needs of primary healthcare clinicians. This process led to three main findings: (1) the use of theory and user-centred design makes it possible to tailor the training program to users’ needs, the effectiveness of this approach being better assessed through qualitative than quantitative techniques; (2) each of the two studied training components appears essential to supporting the adoption of SDM; and (3) training should be tailored to each profession.

### Usefulness of theory-based and user-centered design approaches

The theory-based and user-centered design approach enabled us to identify the strengths and weaknesses of the two training components, and to improve their content, design, navigation, and usability, while solving any technical issues.

The theory-based approach allowed focusing on the known determinants most likely to influence participants’ intention to adopt SDM, a strategy previously recommended in the Behavior Change Wheel Model [52] and in a systematic review of the effectiveness of tailored interventions [54]. Légaré et al. also recommend that the development of Continuing Professional Development activities be based on behaviour change theories, to equip HCPs with the skills needed to change their practice, and ultimately improve clinical performance for the benefit of patients [55]. The use of theory also has the potential to systematically highlight the influences of individual perceptions on the adoption of a targeted behaviour, and thus facilitate the comparison of results obtained with those of other studies interested in implementation science, and ultimately improve the uptake of research findings in practice [56].

Furthermore, the user-centered design approach allowed us to focus on the learners’ perspective of the problem by validating our interpretation of their assessments during the next evaluation round. Several earlier studies have described the benefits of user-centered design to create evidence-based information [30, 31, 57–61], supporting the adoption of these tools in healthcare contexts [62]. However, to the best of our knowledge, no other study to date has used assessments of intention and its determinants within a user-centered design approach to support improvement to tools or training. Future studies should aim to evaluate whether this type of program design improves the adoption of evidence-based practice and patient outcomes.

However, although the use of theory within an iterative user-centered design approach makes it possible to develop high-quality training, it required considerable resources and a continuous commitment from the research team, as previously highlighted in another study [63]. We also found that the semi-structured interviews used during the think-aloud session to optimize the e-learning component were superior to the questionnaires used to evaluate the DBs, despite the fact that the questionnaires used open-text fields that were also analyzed qualitatively. Indeed, the interviews provided greater insight into the weaknesses and strengths of the training components, and allowed us to better adapt the program so as to limit its weaknesses. This coincides with the finding elsewhere that interviews are more robust and provide greater data quality than open-ended survey questions [64].

Findings from this study and from two of our other recent study on user-centered design [31, 65], led to the observation that our systematic approach consisting in modifying a tool every three evaluations is perhaps not optimal. Indeed, if a major problem is discovered after a single evaluation, then modifying this aspect immediately before the next evaluation allows conducting a more useful evaluation in the following round, and optimizing the whole process. This ‘organic’ approach is what is described in the website design literature from which user-centered design originates [66].

The CPD-reaction questionnaire to assess the DBs was also limited in that it was already near to the maximum after a single evaluation round, leaving very little room to measure improvements through subsequent rounds. These results are consistent with those of previous studies that reported a strong intention of clinicians to support women in breast cancer decision-making [67] and a high intention to use DBs in their practice [42]. The examination of the determinants of intention across rounds was therefore more useful than the study of intention to support design of the training program.

### Each training component is important

Overall, our study revealed that each of the two training components was essential for professionals to adopt SDM (Table 7). Firstly, the generic e-learning activity provided knowledge on the theory and practice of SDM. Then, the DBs allowed learners to implement the recommendations in practice, by providing the practical information to discuss specific health matters with their patients. The DBs thus enabled learners to apply the knowledge gained through the e-learning activity to diverse clinical contexts. In the scientific literature, authors are unanimous in observing a lack of structured frameworks to guide the design of e-learning to meet both theoretical and practical needs [68, 69]. DBs, with their simple vocabulary and with their list of services available near to the patient’s home, provide a way to bridge the gap between general knowledge in SDM and its application in specific contexts and for localized populations. In addition, DBs include simple vocabulary and definitions to support professionals in explaining more complex concepts to their patients. They also provide a list of the services available near the patient’s home in order to support implementation of the selected option. This concurs with the findings of a recent integrative review on e-learning to deliver self-management support, that show that incorporating practice and application opportunities improves training effectiveness [68].

### Adapting training to each profession and area

Despite the interprofessional context of care, participants asked that training be adapted to their specific professional role. Indeed, both in the evaluations of DBs and e-learning activity, professionals asked that the clinical examples provided during training match each of their professions. This finding is congruent with the observations that, for continuing professional development to be valued, it must meet the individual needs of professionals, the populations they serve, and the organizations in which they work [70]. This may be because professionals find it more useful and optimal to further their knowledge in areas where they are comfortable. It might also be because they require more incentive to extend their scope of practice, such as a better understanding of the relevance of doing so. This finding converges with conclusions reported in a recent qualitative study to the effect that primary care physicians only read printed educational materials relevant to their patients [71]. Consequently, it might be better to allow participants to select the DBs that coincide with their interests and areas of clinical intervention.

We also found that, for clinicians to consider using the DBs, they should list the health and social services needed to implement the options presented, along with the related contact information. This implies that any decision aid should be tailored to the area where it is distributed.

**Table 7:** Participants’ perceptions of how each program component could facilitate their adoption of shared decision-making

E-learning activity	Decision box
Describes the SDM process Improves knowledge about shared decision-making Sets out the potential benefits of shared decision-making Describes clinical situations where shared decision-making is most relevant Describes situations where SDM should be prioritized and explains how shared decision-making can still be implemented when you’re short of time Explains that SDM requires a discussion around probabilities of experiencing risks associated with each option, and explains why the Decision boxes present statistics Explains how best to present risks, and the evidence underlying these principles	Supports discussion of pros and cons of the options with patients, even for more difficult topics Demonstrates to patients that their values are respected Helps empower patients Offers simpler vocabulary and definitions to explain more complex concepts Supports patients’ understanding of the stakes Helps maintain a therapeutic relationship when a decision threatens this relationship Improves client service Provides up-to-date scientific knowledge needed for professional practice Synthesizes the evidence required to support decision making Improves knowledge of the available options Helps to take ownership of unfamiliar topics and guides patients more effectively toward their preferred option Helps refer patients to the services available near their home in order to support implementation of the selected option

### Determinant of participants’ intention to adopt SDM

In each evaluation round, we observed significant associations between intention and two of its primary determinants, namely beliefs about consequences (or attitude) and social influence. These findings concur with those of an extensive review of the determinants of clinician behaviours, to the effect that said determinants are among those most related to intention [36]. This also confirms previous findings on the influence of these primary determinants on HCPs’ intention to adopt SDM [72], to engage in an interprofessional approach to SDM [73], to participate in a training program in SDM [20], and to use DBs in clinical practice [42]. It is of note that, despite HCPs’ strong intention and the adaptation of tools to users, certain factors external to the training program, including the lack of access to services in some regions, as well as the lack of time, are difficult to control and can still influence the effective use of these tools and the adoption of SDM in practice. In addition, although intention is a useful approximation of actual behavior [74], it may be higher than actual behavior due to a social desirability bias.

## Study limitations

### Despite its comprehensiveness, our study has some limitations

The use of a non-random study sample may also have affected the results by introduction of selection bias. For instance, we may have recruited HCPs with higher intentions to adopt shared decision-making compared to the overall population of professionals who practice in primary care settings. This selection bias could then be responsible for the high intentions we observed in the participants from the start of the study.

The sample sizes were relatively small for the planned quantitative evaluations. Our conclusions were limited by the fact that the quantitative scores showed a ceiling effect. Nevertheless, the qualitative evaluation of each component allowed for a rich description to complement the quantitative results.

The literature indicates that professionals’ intention alone does not always predict SDM adoption [74] or the effective use of SDM tools [75]. It would therefore be interesting for future research to evaluate if professional training programs encourage SDM adoption directly, not only on intention to do so.

We used convenience samples, so the participants who accepted to participate may be different from the general population.

Most participants were physicians and nurses, and none practiced in rural areas. Our conclusions may therefore not apply to other types of professionals, or to those practicing in rural areas. Moreover, our findings may have been different if more social workers had participated in the study, especially since the DB topics concerned social aspects of care. We also acknowledge the high proportion of participants who were women (78%); however, this proportion reflects the overall gender balance among HCPs in Quebec [76].

Some of the comments received during the evaluation of the DBs via the open-text field of the web-based questionnaire were hard to interpret and would have required further discussion with the participants to clarify their opinions.

## Conclusions

A theory-based design approach for continuing professional development interventions on shared decision-making appeared particularly useful to identify the most important determinants of learners’ intentions to use SDM in their clinical practice. A user-centered approach allowed focusing on learners’ perspectives of the problem by validating our initial interpretations of their assessments during the subsequent evaluation round. Participants’ qualitative assessments of the programme provided greater insight into the weaknesses of the training and allowed us to better adapt the program than their quantitative assessments. We also observed a complementarity of the two studied training components, with the e-learning activity providing knowledge on the theory and practice of SDM, and the decision boxes providing the practical information needed to discuss specific health questions with older patients with neurocognitive disorders and their family or friend caregiver. In future research, it would be interesting to compare a group exposed to training tailored using such a theory-based user-centered design approach, to a group exposed to non-tailored training, to assess whether this design approach actually creates a more effective training program to increase adoption of SDM by health professionals. Further research is also needed to evaluate this two-component training program and to highlight the barriers and facilitators for its completion, in order to optimize its implementation and ensure effective uptake and use of SDM.

## Supplementary Information

**Additional file 1.** Participants’ level of intention to adopt shared decision-making and levels of the potential predictors of this intention at baseline and after each evaluation round of the e-learning activity (scale ranges from 1-7).

**Additional file 2.** Participants’ level of intention to use what they learned through the DBs to explain the pros and cons of health options to patients, and levels of the potential predictors of this intention at each evaluation round (scale ranges from 1-7).

## Data Availability

The datasets used and/or analysed during the current study are available from the corresponding author on reasonable request.

## Abbreviations

DB: Decision box
HCP: Healthcare professional
IAM: Information Assessment Method
SDM: Shared Decision Making
TAM-2: Technology Acceptance Model
TPB: Theory of Planned Behaviour
