# Supplementary material for: User-centered and theory-based design of a professional training program on shared decision-making with older adults living with neurocognitive disorders: a mixed-methods study
Source: BMC Med Inform Decis Mak. 2021 Feb 17;21:59. doi: 10.1186/s12911-021-01396-y (PMC7888116; doi:10.1186/s12911-021-01396-y)
Supplement: Supplementary file 1 — Additional file 1. Participants’ level of intention to adopt shared decision-making and levels of the potential predictors of this intention at baseline and after each evaluation round of the e-learning activity (scale ranges from 1-7). [file 12911_2021_1396_MOESM1_ESM.docx]

# Additional file 1: Participants’ level of intention to adopt shared decision making and levels of the potential predictors of this intention at baseline and after each evaluation round of the e-learning activity (scale ranges from 1-7).

| **Scale, Domain** |  | **Mean (± SD)**  **[Range]** | | | |
| --- | --- | --- | --- | --- | --- |
|  | **Baseline**  **n=16** | **Round #1**  **n=5** | **Round #2**  **n=5** | **Round #3**  **n=6** | **All Rounds**  **n=16** |
| **CPD REACTION** |  |  |  |  |  |
| Intention | 6.5 (0.62) | 6.9 (0.15) | 7.0 (0.00) | 6.6 (0.50) | 6.8 (0.36) |
|  | [5.0 – 7.0] | [6.7 – 7.0] | [7.0 – 7.0] | [5.7 – 7.0] | [5.7 – 7.0] |
| Beliefs about Consequences | 6.4 (0.68) | 6.8 (0.30) | 6.9 (0.15) | 6.6 (0.54) | 6.8 (0.39) |
|  | [4.7 – 7.0] | [6.3 – 7.0] | [6.7 – 7.0] | [5.7 – 7.0] | [5.7 – 7.0] |
| Social Influence | 5.6 (0.76) | 6.0 (0.75) | 5.7 (0.45) | 5.7 (0.50) | 5.8 (0.56) |
|  | [3.9 – 7.0] | [4.7 – 6.7] | [5.0 – 6.2] | [4.7– 6.2] | [4.7 – 6.7] |
| Beliefs about Capabilities | 6.0 (0.67) | 6.5 (0.45) | 6.5 (0.38) | 6.3 (0.47) | 6.4 (0.42) |
|  | [5.0 – 7.0] | [6.0 – 7.0] | [6.6 – 7.0] | [5.7 – 7.0] | [5.7 – 7.0] |
| Moral Norm | 6.7 (0.43) | 7.0 (0.00) | 7.0 (0.00) | 6.7 (0.52) | 6.9 (0.34) |
|  | [5.7 – 7.0] | [7.0 – 7.0] | [7.0 – 7.0] | [6.0 – 7.0] | [6.0 – 7.0] |
| **TAM-2** |  |  |  |  |  |
| Usefulness | NA | 6.5 (0.40) | 6.3 (0.48) | 6.5 (0.49) | 6.4 (0.44) |
|  |  | [6.0 – 7.0] | [5.8 – 7.0] | [5.8 – 7.0] | [5.8 – 7.0] |
| Ease of Use | NA | 6.6 (0.34) | 6.1 (0.52) | 6.1 (0.65) | 6.3 (0.54) |
|  |  | [6.0 – 6.8] | [5.5 – 6.8] | [5.3 – 7.0] | [5.3 – 7.0] |

NA=not applicable
