# Supplementary material for: User-centered and theory-based design of a professional training program on shared decision-making with older adults living with neurocognitive disorders: a mixed-methods study
Source: BMC Med Inform Decis Mak. 2021 Feb 17;21:59. doi: 10.1186/s12911-021-01396-y (PMC7888116; doi:10.1186/s12911-021-01396-y)
Supplement: Supplementary file 2 — Additional file 2. Participants’ level of intention to use what they learned through the DBs to explain the pros and cons of health options to patients, and levels of the potential predictors of this intention at each evaluation round (scale ranges from 1-7). [file 12911_2021_1396_MOESM2_ESM.docx]

# Additional file 2: Participants’ level of intention to use what they learned through the DBs to explain the pros and cons of health options to patients, and levels of the potential predictors of this intention at each evaluation round (scale ranges from 1-7).

| **Scale, Domain** | **Mean (± SD)**  **[Range]** | | | | |
| --- | --- | --- | --- | --- | --- |
|  | **Round #1**  **n=21** | **Round #2**  **n=17** | **Round #3**  **n=18** | **Round #4**  **n=108** | **All Rounds**  **n=164** |
| **CPD REACTION** |  |  |  |  |  |
| Intention | 5.5 (0.97) | 5.5 (1.10) | 5.6 (1.12) | 5.8 (1.29) | 5.7 (1.22) |
|  | [3.3; 7.0] | [2.0; 6.7] | [3.3; 7.0] | [1.0; 7.0] | [1.0; 7.0] |
| Beliefs about Consequences | 5.3 (0.88) | 5.7 (0.74) | 5.4 (1.03) | 5.8 (1.10) | 5.7 (1.05) |
|  | [3.3; 6.7] | [3.7; 6.3] | [2.7; 6.3] | [1.0; 7.0] | [1.0; 7.0] |
| Social Influence | 4.2 (0.88) | 4.2 (0.72) | 4.2 (0.97) | 4.7 (0.94) | 4.5 (0.94) |
|  | [2.7; 5.7] | [3.0; 5.3] | [2.7; 5.3] | [2.0; 7.0] | [2.0; 7.0] |
| Beliefs about Capabilities | 5.4 (0.90) | 5.6 (0.75) | 5.5 (0.86) | 5.1 (0.90) | 5.2 (0.90) |
|  | [3.3; 7.0] | [3.7; 6.7] | [4.0; 6.7] | [3.0; 7.0] | [3.0; 7.0] |
| Moral Norm | 6.0 (0.98) | 6.1 (0.78) | 6.1 (0.75) | 6.5 (0.87) | 6.4 (0.88) |
|  | [3.7; 7.0] | [4.3; 7.0] | [4.0; 7.0] | [2.0; 7.0] | [2.0; 7.0] |
| **TAM-2** |  |  |  |  |  |
| Usefulness | 5.7 (0.67) | 5.6 (0.89) | 5.6 (1.24) | 6.0 (0.81) | 5.9 (0.87) |
|  | [3.8; 7.0] | [4.0;7.0] | [3.3; 7.0] | [3.5; 7.0] | [3.3; 7.0] |
| Ease of Use | 5.7 (0.73) | 6.0 (0.69) | 5.8 (1.06) | 5.8 (1.01) | 5.8 (0.95) |
|  | [3.5; 7.0] | [4.5; 7.0] | [3.5; 7.0] | [2.0; 7.0] | [4.0; 7.0] |
| **Satisfaction** | 4.0 (0.80) | 4.1 (0.66) | 4.0 (0.85) | 4.3 (0.72) | 4.2 (0.74) |
|  | [2.0; 5.0] | [3.0; 5.0] | [2.0; 5.0] | [2.0; 5.0] | [2.0; 5.0] |
